# Pulmonary microbiome and metabolome signatures associate with chemotherapy response in lung cancer patients

**DOI:** 10.3389/fmicb.2025.1604999

**Published:** 2025-06-13

**Authors:** Xuehang Jin, Lvjun Zhang, Chiqing Ying, Kailing Pan, Dan Zhu

**Affiliations:** ^1^Department of Respiratory and Critical Care Medicine, Affiliated Jinhua Hospital, Zhejiang University School of Medicine, Jinhua, China; ^2^Department of Central Laboratory, Affiliated Jinhua Hospital, Zhejiang University School of Medicine, Jinhua, China

**Keywords:** lung cancer, metabolite, microbiota, chemotherapy, biomarker

## Abstract

**Background:**

Lung cancer is a leading cause of cancer-related mortality worldwide, with chemotherapy response varying significantly among patients. Emerging evidence suggests that the pulmonary microbiota and metabolome may influence treatment outcomes, but their roles remain unclear.

**Methods:**

This study enrolled 25 lung cancer patients undergoing chemotherapy, categorized into chemotherapy-sensitive (*n* = 15) and chemotherapy-insensitive (*n* = 10) groups. Bronchoalveolar lavage fluid (BALF) was collected for 16S rDNA sequencing and untargeted metabolomics (LC-MS). Serum bile acids were also analyzed.

**Results:**

The study identified 92 significantly altered metabolites in BALF between the two groups. Trans-urocanate showed the highest increase, while phenylalanylphenylalanine exhibited the greatest decrease in sensitive patients. Key metabolic pathways, including ABC transporters, glutathione metabolism, and bile acid biosynthesis, were enriched. Microbiome analysis revealed differential abundances of specific bacterial genera, particularly increased Caulobacter and decreased Acinetobacter in sensitive patients. Notably, serum levels of four bile acids (chenodeoxycholic acid, cholic acid, deoxycholic acid, and ursodeoxycholic acid) were significantly elevated in chemotherapy-sensitive patients, demonstrating good predictive value with AUCs ranging from 0.633 to 0.830.

**Conclusion:**

The study highlights distinct microbial and metabolic signatures associated with chemotherapy response, suggesting potential biomarkers for personalized therapy.

## 1 Introduction

Lung cancer remains one of the most common and deadly malignancies globally, contributing significantly to cancer-related morbidity and mortality (Miller et al., 2019). Despite advances in early detection and treatment, the prognosis for lung cancer patients remains poor, particularly in advanced stages. Chemotherapy, a cornerstone of lung cancer treatment, has shown efficacy in improving survival rates and alleviating symptoms. However, patient responses vary widely, with some benefiting significantly while others show minimal or no clinical improvement (Boyerinas et al., 2015; Gettinger et al., 2015). This variability underscores the need for reliable biomarkers to predict chemotherapy efficacy and guide personalized treatment strategies.

Recent studies have highlighted the complex interactions between the pulmonary microbiota and lung cancer pathogenesis, progression, and treatment response. Once considered sterile, the lung is now recognized as a dynamic ecosystem harboring diverse microbial communities. Alterations in these communities have been linked to lung cancer development, immune modulation, and therapy resistance (Yang et al., 2023; Zhao et al., 2021; King, 2024). Emerging evidence suggests that specific microbial signatures may influence chemotherapy efficacy by modulating the tumor microenvironment, immune responses, and drug metabolism (Xue et al., 2023). Analyzing microbial profiles in bronchoalveolar lavage fluid (BALF) from lung cancer patients could provide insights into microbial determinants of treatment response and identify novel predictive biomarkers.

The lung’s metabolic landscape also plays a critical role in cancer biology and treatment outcomes. Metabolites, as end products of cellular processes, reflect the interplay between host metabolism and microbial activity. Dysregulated metabolic pathways, such as glycolysis, amino acid metabolism, and lipid metabolism, are hallmarks of cancer and have been associated with tumor progression, metastasis, and chemotherapy resistance (Yoo et al., 2020; Faubert et al., 2020). Furthermore, the pulmonary microbiota can produce bioactive metabolites that influence host immunity and drug efficacy. By examining metabolic signatures in BALF, we can identify metabolites associated with chemotherapy response, complementing microbial biomarkers for treatment prediction. Integrating microbial and metabolic profiling holds great promise for advancing precision medicine in lung cancer chemotherapy.

## 2 Materials and methods

### 2.1 Study cohorts

This study included a cohort of 25 patients diagnosed with lung cancer, all of whom underwent bronchoscopy at the Department of Respiratory and Critical Care Medicine, Affiliated Jinhua Hospital, Zhejiang University School of Medicine. The patients were admitted with space-occupying lesions in the lung between January 2024 and July 2024. BALF, serum, and clinical data were collected from each participant. The inclusion criteria for the study were: (1) initial patient visit, (2) subsequent confirmed diagnosis of lung cancer, and (3) performance of bronchoscopy and collection of BALF at our institution, (4) follow-up in our hospital for 4–6 times of chemotherapy. The exclusion criteria were as follows: (1) history of cancer, (2) antibiotic use within the preceding 3 months, and (3) presence of co-infection, immunodeficiency, or liver and kidney dysfunction.

Compete response (CR) and partial response (PR) were defined as chemo-sensitive patients. Stable disease (SD) and progressed disease (PD) were defined as chemo-insensitive patients.

All participants provided informed consent prior to inclusion in the study. The research adhered to the principles outlined in the Declaration of Helsinki and received approval from the Institutional Review Board of Affiliated Jinhua Hospital, Zhejiang University School of Medicine (Ethics Approval Number: AL-JHYY202437).

### 2.2 BALF collection

BALF was collected from affected pulmonary lobes using sterile tubes under standardized protocols to minimize oral contamination. After centrifugation (15,000 g, 30 min, 4°C), supernatants and cell pellets were stored at −80°C for metabolomics and 16S rDNA sequencing, respectively.

### 2.3 16S rDNA high-throughput sequencing and data processing

Genomic DNA was extracted from BALF pellets, and the V3–V4 regions of the 16S rRNA gene were amplified using standard primers (341F/806R). Libraries were prepared with dual-indexed barcodes and sequenced on the Illumina MiSeq platform (2 × 250 bp).

### 2.4 Untargeted metabolomics by LC-MS

BALF metabolites were analyzed using a UHPLC-Q Exactive HF-X system (Thermo Scientific) with an ACQUITY UPLC HSS T3 column (2.1 × 100 mm, 1.8 μm). Mobile phases consisted of (A) 0.1% formic acid in water and (B) acetonitrile. Gradient elution: 0–12 min, 5–95% B; 12–14 min, 95% B; 14–14.1 min, 95–5% B; 14.1–16 min, 5% B. Mass spectrometry operated in positive/negative ion modes with spray voltage ±3.8 kV and resolution 70,000.

### 2.5 Serum bile acid profile detection

Bile acids were quantified utilizing liquid chromatography-tandem mass spectrometry, which included the measurement of five free bile acids—cholic acid (CA), chenodeoxycholic acid (CDCA), deoxycholic acid (DCA), lithocholic acid (LCA), and ursodeoxycholic acid (UDCA)—alongside 10 conjugated bile acids: glycocholic acid (GCA), glycochenodeoxycholic acid (GCDCA), glycodeoxycholic acid (GDCA), glycolithocholic acid (GLCA), glycoursodeoxycholic acid (GUDCA), taurocholic acid (TCA), taurochenodeoxycholic acid (TCDCA), taurodeoxycholic acid (TDCA), taurolithocholic acid (TLCA), and tauroursodeoxycholic acid (TUDCA). This analytical procedure was performed by Dian Diagnostic Technology Co., Ltd.

### 2.6 Statistical analysis

To compare the abundances of individual taxa between two groups, the STAMP software was employed, while LEfSe was utilized for the quantitative analysis of biomarkers. Differences in microbial communities were evaluated using ANOSIM and ADONIS, based on Bray–Curtis dissimilarity matrices. Additional analyses included principal component analysis (PCA) and multivariate statistical assessments with SIMCA 14.1, Pearson correlation analysis via CytoScape 3.5.1, and KEGG pathway analysis conducted using R 3.5.1. Statistical significance was denoted by *p*-values, with * indicating *p* < 0.05, ** indicating *p* < 0.01, and *** indicating *p* < 0.001.

## 3 Results

### 3.1 Patients characteristics

In this study, we included 25 patients diagnosed with lung cancer via bronchoscopy and subsequently treated with chemotherapy at our hospital. These patients were categorized into two groups: a chemotherapy-sensitive group comprising 15 individuals and a chemotherapy-insensitive group consisting of 10 individuals. BALF was collected from all participants, and both microbiological and metabolomic analyses were conducted. The baseline characteristics of the two patient groups in the study cohort were detailed in Table 1. Our analysis revealed no significant differences between the groups in terms of age, sex, pathological type, liver function (TBA, ALT, AST, and TBIL), or tumor markers (CEA, CA199, CA125, SCC, CK19, NSE, and ProGRP).

**Table 1:** Patient characteristics at baseline (*n* = 25).

Characteristic	Chemotherapy-sensitive	Chemotherapy-insensitive	*p*-value
Number	15	10	
Age, mean ± SD, years	63.9 ± 10.1	65.3 ± 10.1	0.545
Sex			0.668
Male, *n* (%)	12 (80.0)	7 (70.0)	
Female, *n* (%)	3 (20.0)	3 (30.0)	
Histology			0.206
Adenocarcinomas, *n* (%)	5 (33.3)	6 (60.0)	
*Squamous cell* carcinomas, *n* (%)	7 (46.7)	2 (20.0)	
*Small cell* lung cancer, *n* (%)	3 (20.0)	2 (20.0)	
TBA, mean ± SD	6.4 ± 4.6	6.4 ± 4.6	0.271
ALT, mean ± SD	25.8 ± 57.8	24.1 ± 54.1	0.288
AST, mean ± SD	26.2 ± 33.2	25.3 ± 31.1	0.312
TBIL, mean ± SD	13.4 ± 4.8	13.5 ± 6.4	0.297
CEA, mean ± SD	41.0 ± 164.3	36.2 ± 153.2	0.189
CA199, mean ± SD	55.0 ± 156.7	49.2 ± 147.0	0.418
CA125, mean ± SD	58.8 ± 79.2	53.9 ± 75.8	0.064
SCC, mean ± SD	2.1 ± 3.7	2.0 ± 3.5	0.203
CK19, mean ± SD	11.2 ± 18.3	11.0 ± 17.1	0.598
NSE, mean ± SD	28.7 ± 62.6	27.8 ± 58.5	0.245
ProGRP, mean ± SD	498.9 ± 1362.3	443.1 ± 1274.5	0.687

### 3.2 Differential metabolites in BALF between chemotherapy-sensitive and chemotherapy-insensitive patients

**Figure 1:**
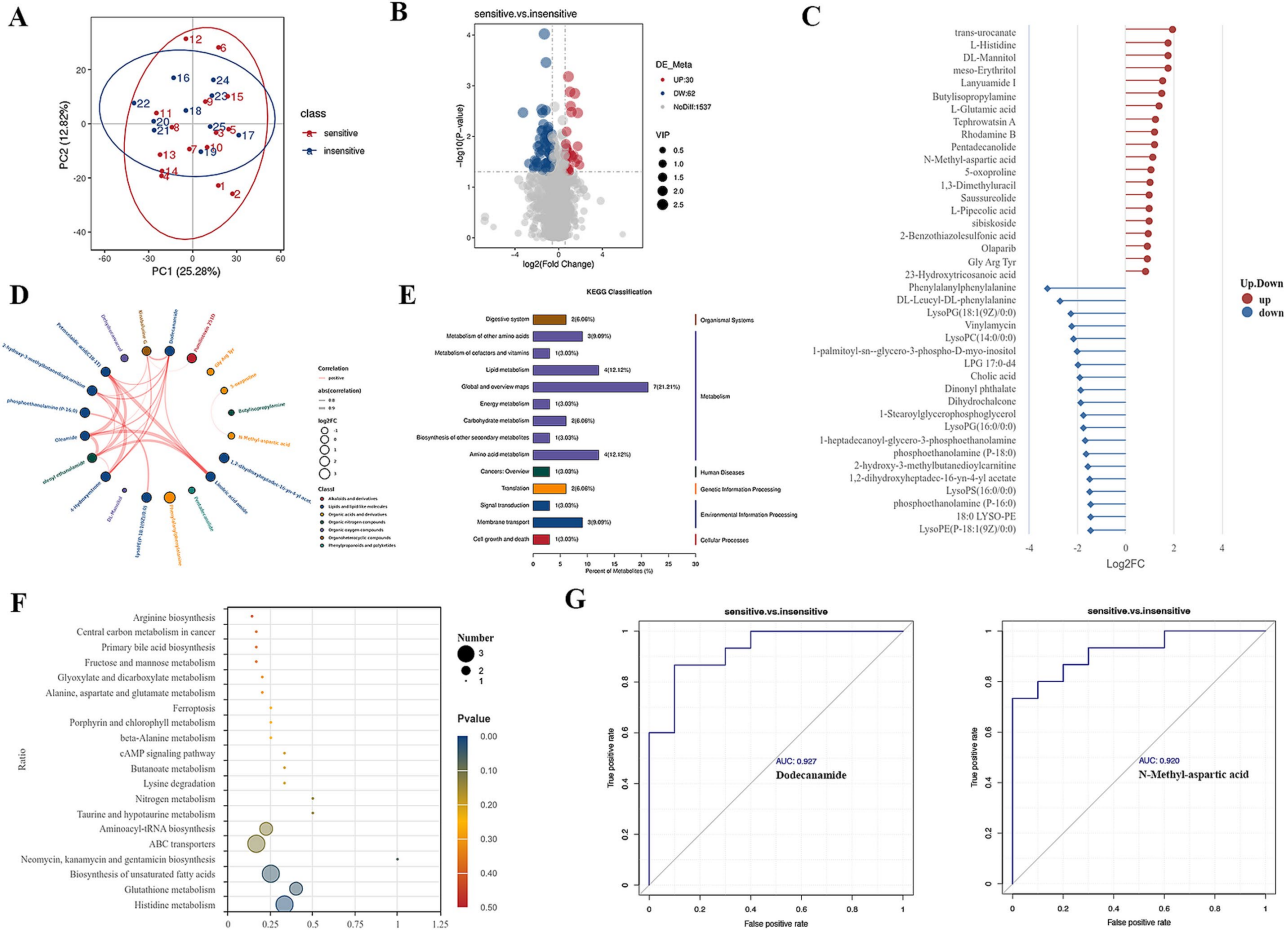
Differential metabolites in BALF between chemotherapy-sensitive and chemotherapy-insensitive patients. **(A)** Principal component analysis (PCA) of the differentially metabolites. **(B)** The volcano map of differential metabolites. **(C)** Significant differentially metabolites with a log2fold change. **(D)** Chord diagram showing the relationship between correlated differential metabolites. **(E,F)** Enriched pathways between the two groups. **(G)** ROC curve was used to investigate the diagnostic value of representative differential metabolites.

Metabolites are crucial in the onset, progression, and therapeutic response of tumors, particularly in lung cancer. Alterations in alveolar metabolites can directly influence the efficacy of chemotherapy. To elucidate the relationship between lung metabolites and chemotherapy sensitivity in lung cancer, we categorized the enrolled patients into two cohorts. Comparative analyses indicated no statistically significant differences between the groups concerning gender, age, histological type, and laboratory results (*p* > 0.05). principal component analysis (PCA) and partial least squares discriminant analysis (PLS-DA) (Figure 1A; Supplementary Figure 1A), along with their respective permutation tests (Supplementary Figure 1B), identified a distinct set of differential metabolites associated with the two groups.

We utilized ultra-high-performance liquid chromatography coupled with quadrupole time-of-flight mass spectrometry (UHPLC-Q-TOF MS) to perform high-resolution untargeted metabolomics analysis for metabolite identification. This methodology facilitated the detection of 1,629 metabolites in the positive ion mode and negative ion mode. To discern differential metabolites in the BALF between the two groups of lung cancer patients, we identified significant differences in metabolite levels based on the variable importance in projection (VIP) score, employing a significance threshold of *p* < 0.05 and a fold change greater than 1.5. This analysis resulted in the identification of 92 metabolites, of which 32 were significantly elevated and 60 were significantly decreased in the chemotherapy-sensitive group (Figure 1B and Table 2). In the chemotherapy-sensitive group, trans-urocanate had the highest increase, while Phenylalanylphenylalanine had the greatest decrease among the abundant metabolites (Figure 1C).

**Table 2:** Differential metabolites in BALF between chemotherapy-sensitive and chemotherapy-insensitive patients.

Name	Formula	Molecular weight	log2FC	*p*-value	ROC
Dodecanamide	C_12_H_25_NO	199.19	−1.31	<0.001	0.93
Pumiliotoxin 251D	C_16_H_29_NO	251.22	−1.16	<0.001	0.91
Gly Arg Tyr	C_17_H_26_N_6_O_5_	394.20	0.89	0.001	0.88
5-Oxoproline	C_5_H_7_NO_3_	129.04	1.04	0.001	0.86
Butylisopropylamine	C_7_H_17_N	115.14	1.47	0.001	0.83
N-Methyl-aspartic acid	C_5_H_9_NO_4_	147.05	1.09	0.003	0.92
1,2-Dihydroxyheptadec-16-yn-4-yl acetate	C_19_H_34_O_4_	326.25	−1.53	0.003	0.85
Linoleic acid amide	C_18_H_33_NO	279.26	−1.20	0.003	0.88
Pentadecanolide	C_15_H_28_O_2_	240.21	1.16	0.003	0.81
Phenylalanylphenylalanine	C_18_H_20_N_2_O_3_	312.15	−3.26	0.003	0.84
LysoPE(P-18:1(9Z)/0:0)	C_23_H_46_NO_6_P	463.31	−1.49	0.003	0.83
DL-mannitol	C_6_H_14_O_6_	182.08	1.74	0.003	0.84
4-Hydroxyestrone	C_18_H_22_O_3_	286.16	−1.39	0.006	0.83
Oleoyl ethanolamide	C_20_H_39_NO_2_	325.30	−0.95	0.007	0.83
Oleamide	C_18_H_35_NO	281.27	−0.96	0.007	0.83
Phosphoethanolamine (P-16:0)	C_21_H_44_NO_6_P	437.29	−1.49	0.007	0.81
2-Hydroxy-3-methylbutanedioylcarnitine	C_12_H_21_NO_7_	291.13	−1.58	0.008	0.80
Petroselaidic acid (C18-1 T)	C_18_H_34_O_2_	282.26	−1.01	0.008	0.83
Dehydrocarvacrol	C_10_H_12_O	148.09	0.72	0.008	0.78
Kinabalurine G	C_11_H_21_NO_2_	199.16	−0.64	0.009	0.79
LysoPE(22:6(4Z,7Z,10Z,13Z,16Z,19Z)/0:0)	C_27_H_44_NO_7_P	525.29	−0.92	0.009	0.84
(E)-Nerolidol acetate	C_17_H_28_O_2_	264.21	−1.17	0.010	0.80
Hemi-babim	C_16_H_14_N_6_	290.13	−1.28	0.011	0.82
1-Heptadecanoyl-glycero-3-phosphoethanolamine	C_22_H_46_NO_7_P	467.30	−1.71	0.011	0.81
2-Methyl-5-(8,11-pentadecadienyl)-1,3-benzenediol	C_22_H_34_O_2_	330.26	−1.07	0.011	0.78
Tashironin	C_22_H_26_O_6_	386.17	−0.96	0.012	0.79
PE(18:1(9Z)/0:0)	C_23_H_46_NO_7_P	479.30	−1.31	0.013	0.79
1,2,3-Tris(1-ethoxyethoxy)propane	C_15_H_32_O_6_	308.22	−0.76	0.014	0.78
Coniine	C_8_H_17_N	127.14	0.66	0.014	0.83
Estrane	C_18_H_30_	246.23	−1.15	0.015	0.85
LysoPG(16:0/0:0)	C_22_H_45_O_9_P	484.28	−1.76	0.015	0.78
Meso-erythritol	C_4_H_10_O_4_	122.06	1.74	0.016	0.79
Dinonyl phthalate	C_26_H_42_O_4_	418.31	−1.90	0.016	0.79
Geranyl benzoate	C_17_H_22_O_2_	258.16	−1.28	0.017	0.81
3,7,8,15-Scirpenetetrol	C_15_H_22_O_6_	298.14	0.73	0.017	0.75
N6,O2′-Dimethyladenosine	C_12_H_17_N_5_O_4_	295.13	−0.85	0.017	0.78
Pseudo-anisatin	C_15_H_22_O_6_	298.14	0.75	0.017	0.75
Schizanthine E	C_26_H_38_N_2_O_7_	490.27	−0.68	0.019	0.75
LysoPS(16:0/0:0)	C_22_H_44_NO_9_P	497.28	−1.52	0.019	0.77
Tuberostemospironine	C_13_H_19_NO_4_	253.13	0.73	0.019	0.78
N-Lauroyl glutamic acid	C_17_H_31_NO_5_	329.22	−0.64	0.020	0.76
1-Stearoylglycerophosphoglycerol	C_24_H_49_O_9_P	512.31	−1.78	0.020	0.78
LysoPE(20:4(8Z,11Z,14Z,17Z)/0:0)	C_25_H_44_NO_7_P	501.29	−0.93	0.021	0.77
1,3-Dimethyluracil	C_6_H_8_N_2_O_2_	140.06	0.99	0.021	0.79
Hexadecanoylpyrrolidine	C_20_H_39_NO	309.30	−0.86	0.022	0.79
Kirenol	C_20_H_34_O_4_	338.25	−0.84	0.022	0.75
Phosphoethanolamine (P-18:0)	C_23_H_48_NO_6_P	465.32	−1.66	0.022	0.75
4,4-Dimethoxy-2-butanone	C_6_H_12_O_3_	132.08	0.64	0.023	0.81
L-Glutamic acid	C_5_H_9_NO_4_	147.05	1.38	0.023	0.77
Rhodamine B	C_28_H_31_ClN_2_O_3_	478.20	1.18	0.024	0.71
2-Benzothiazolesulfonic acid	C_7_H_5_NO_3_S_2_	214.97	0.90	0.025	0.83
Tephrowatsin A	C_22_H_26_O_4_	354.18	1.21	0.025	0.78
L-Histidine	C_6_H_9_N_3_O_2_	155.07	1.75	0.028	0.71
Gamma-LINOLENIC acid	C_18_H_30_O_2_	278.22	−0.66	0.029	0.75
4,4′-Dimethoxychalcone	C_17_H_16_O_3_	268.11	−0.63	0.029	0.74
LysoPC(14:0/0:0)	C_22_H_46_NO_7_P	467.30	−2.19	0.029	0.81
1-Palmitoyl-sn--glycero-3-phospho-D-myo-inositol	C_25_H_49_O_12_P	572.30	−2.05	0.030	0.79
Isoketocamphoric acid	C_10_H_16_O_5_	216.10	0.67	0.030	0.75
Eupomatenoid 5	C_19_H_18_O_3_	294.13	−0.72	0.031	0.77
Cis-11-Eicosenoic acid	C_20_H_38_O_2_	310.29	−1.02	0.032	0.81
N-(3-amino-3-oxopropyl)-L-valine	C_8_H_16_N_2_O_3_	188.12	0.59	0.032	0.77
Cinnamyl phenylacetate	C_17_H_16_O_2_	252.12	−0.97	0.033	0.75
Lanyuamide I	C_18_H_31_NO_2_	293.24	1.52	0.033	0.75
Dihydrochalcone	C_15_H_14_O	210.10	−1.89	0.033	0.77
Dodeca-4,7,10-trienoylcarnitine	C_19_H_31_NO_4_	337.23	−1.18	0.033	0.79
LPG 17:0-d4	C_23_H_47_O_9_P	498.30	−2.01	0.033	0.74
N6-Carboxyethyllysine	C_9_H_18_N_2_O_4_	218.13	0.66	0.033	0.72
Docosahexaenoic acid	C_22_H_32_O_2_	328.24	−1.21	0.034	0.77
DL-Leucyl-DL-phenylalanine	C_15_H_22_N_2_O_3_	278.16	−2.73	0.034	0.73
Kaurenoic acid	C_20_H_30_O_2_	302.22	−0.71	0.035	0.76
Trans-urocanate	C_6_H_6_N_2_O_2_	138.04	1.92	0.037	0.72
Cholic acid	C_24_H_40_O_5_	408.29	−1.92	0.039	0.73
Vinylamycin	C_26_H_43_N_3_O_6_	493.32	−2.28	0.040	0.79
Cucurbitacin E	C_32_H_44_O_8_	556.30	−1.14	0.041	0.74
Hexanoylglutamine	C_11_H_20_N_2_O_4_	244.14	−0.79	0.041	0.72
23-Hydroxytricosanoic acid	C_23_H_46_O_3_	370.34	0.79	0.042	0.77
Abafungin	C_21_H_22_N_4_OS	378.15	−0.83	0.042	0.71
Penciclovir	C_10_H_15_N_5_O_3_	253.12	−0.95	0.043	0.73
Bidisomide	C_22_H_34_ClN_3_O_2_	407.23	−1.19	0.043	0.69
LysoPG(18:1(9Z)/0:0)	C_24_H_47_O_9_P	510.30	−2.31	0.043	0.71
Saussureolide	C_15_H_22_O_6_	298.14	0.96	0.043	0.73
LysoPE(20:1(11Z)/0:0)	C_25_H_50_NO_7_P	507.33	−1.45	0.044	0.74
Olaparib	C_24_H_23_FN_4_O_3_	434.18	0.89	0.045	0.71
8-Methoxycoumarin	C_10_H_8_O_3_	176.05	−0.65	0.045	0.70
1-Pentanone, 3-hydroxy-1,5-diphenyl-	C_17_H_18_O_2_	254.13	−1.21	0.046	0.71
18:0 LYSO-PE	C_23_H_48_NO_7_P	481.32	−1.49	0.047	0.75
16:0 LYSO-PE	C_21_H_44_NO_7_P	453.29	−1.16	0.049	0.73
LysoPE(18:2w6/0:0)	C_23_H_44_NO_7_P	477.29	−1.00	0.049	0.72
Sibiskoside	C_16_H_24_O_8_	344.15	0.94	0.049	0.72
Nb-Stearoyltryptamine	C_28_H_46_N_2_O	426.36	0.73	0.049	0.75
L-Pipecolic acid	C_6_H_11_NO_2_	129.08	0.95	0.049	0.69
CPA(16:0/0:0)	C_19_H_37_O_6_P	392.23	−0.81	0.050	0.75

log2FC, fold change of gene expression in log2 scale. Positive log2FC indicates upregulated metabolites in chemotherapy-sensitive patients; negative log2FC indicates downregulated metabolites in chemotherapy-sensitive patients.

We used STITCH to build a metabolic network of correlated differential metabolites to examine their interactions. Figure 1D shows that all these metabolites are interconnected, either directly or indirectly. Strong interactions were observed between the linoleic acid amide, petroselaidic acid (C18-1 T), oleamide, and oleoyl ethanolamide (all interaction scores >0.9).

KEGG pathway enrichment analysis was performed by Fisher’s exact test to identify metabolic and signal transduction pathways significantly associated with chemo sensitivity in lung cancer patients. The results showed that ABC transporters, biosynthesis of unsaturated fatty acids, glutathione metabolism, and histidine metabolism pathways were significantly enriched (Figures 1E,F).

Our GSEA analysis of differential metabolites revealed significant enrichment in metabolic pathways, biosynthesis of amino acids, ABC transporters, and bile secretion pathways (Supplementary Figures 1C–F). Furthermore, AUC (area under the ROC curve) analysis revealed several metabolites with high sensitivity and specificity in differentiating lung cancer patients with chemotherapy sensitive from those with insensitive, including dodecanamide (AUC = 0.927), N-methyl-aspartic acid (AUC = 0.920) (Figure 1G).

### 3.3 Differential airway microbiome in BALF between chemotherapy-sensitive and chemotherapy-insensitive patients

**Figure 2:**
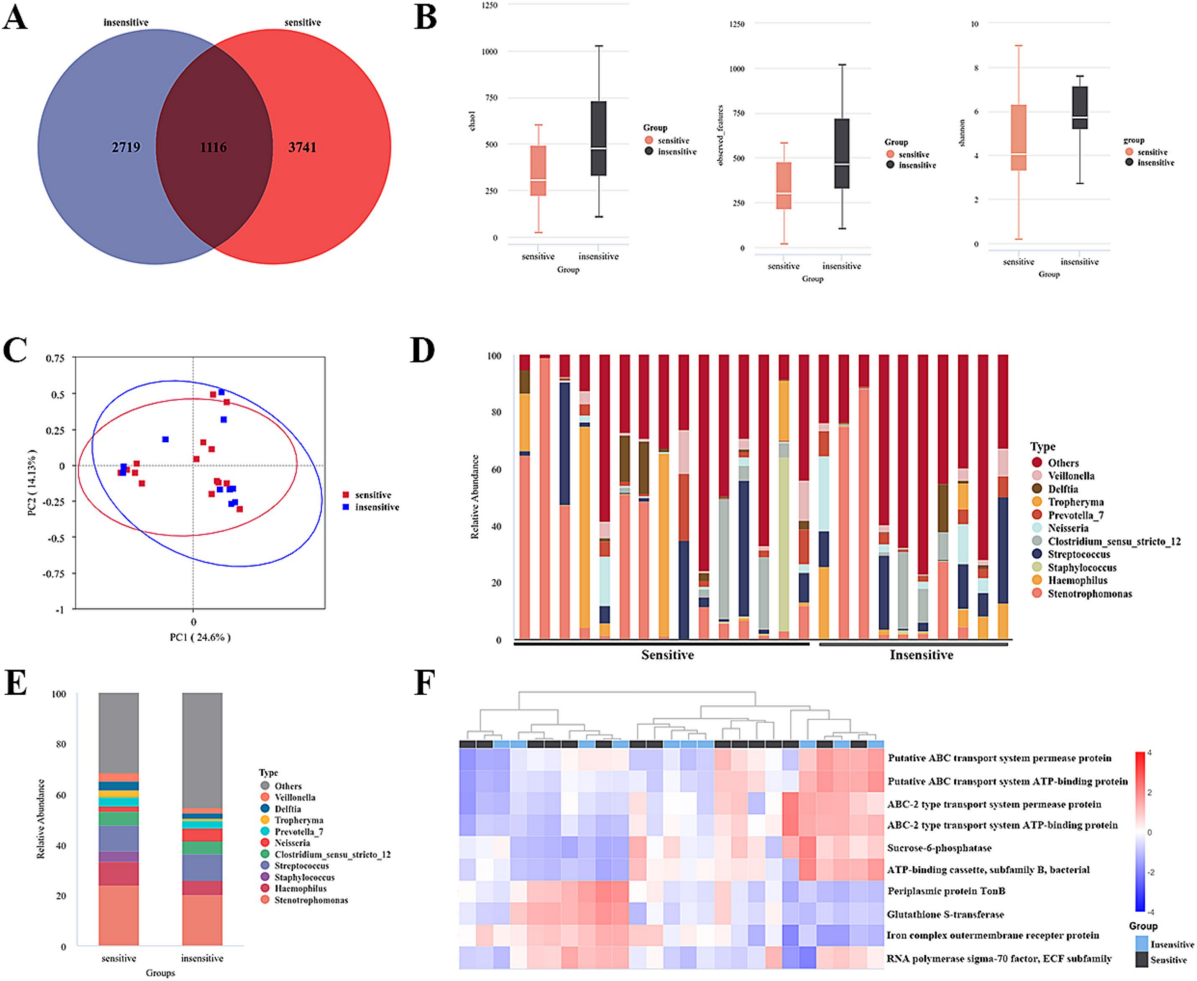
Differential microbiome in BALF between chemotherapy-sensitive and chemotherapy-insensitive patients. **(A)** The differential abundance analysis between two groups was compared using a Venn plot. **(B)** α-diversity between two groups was measured by the Chao, Shannon, and observed features. **(C)** PLS-DA was conducted to compare the overall similarities in the bacterial taxonomy based on β-diversity. **(D)** The relative frequency of top abundant taxa in each patient at the genus level. **(E)** The relative frequency of top abundant taxa in each group at the genus level. **(F)** Predicted functions of lung cancer bacteria were analyzed using PICRUSt2.

In the present microbiome investigation, we successfully performed PCR amplification of the 16S rDNA gene, specifically targeting the V3–V4 regions, across all collected samples. The Venn diagram depicted in Figure 2A, derived from the ASV table data, visually represents the quantity of shared and unique ASVs for each group. The analysis revealed that the two groups shared 1,116 ASVs, with the sensitive group possessing 3,741 ASVs and the insensitive group possessing 2,719 ASVs.

Three diversity indices—Chao 1, Shannon, and observed species—were employed to assess α-diversity between the groups. The analysis revealed no significant differences, indicating that there was no notable alteration in lung microbial community diversity between the two groups (Figure 2B). To further examine the similarities in bacterial communities between the groups, a principal coordinates analysis (PCoA) plot was generated using unweighted UniFrac distances at the operational taxonomic unit (OTU) level. The results demonstrated no discernible separation between the groups, suggesting that the primary composition of the lung microbiome in the cohort with lung cancer and distant metastasis remained largely unchanged (Figure 2C).

A subsequent analysis of the compositional disparities in the pulmonary microbiota between the two patient cohorts demonstrated that, although numerous taxa were shared, their relative abundances exhibited significant differences. Notably, substantial variations were observed in the proportions of Stenotrophmonas, Streptococcus, Haemophilus, Pseudomonas, Neisseria, Veillonella, Tropheryma, and Staphylococcus between the two groups, as illustrated in Figures 2D,E. Moreover, a *t*-test highlighted microecological variations between the two groups, showing a notable increase in the Caulobacter (*p* = 0.003), CL500-29_marine_group (*p* = 0.002), [Eubacterium]_eligens_group (*p* = 0.0046) and decrease in the Acinetobacter (*p* = 0.031), Abiotrophia (*p* = 0.010) genus within the alveolar lavage fluid of sensitive patients (Table 3).

**Table 3:** Differential microbiome in BALF between chemotherapy-sensitive and chemotherapy-insensitive patients.

Microbiome (genus)	LogFC	*p*-values
Acinetobacter	−1.503	0.031
Abiotrophia	−1.365	0.010
Caulobacter	1.218	0.003
CL500-29_marine_group	1.207	0.002
[Eubacterium]_eligens_group	1.006	0.046

log2FC, fold change of gene expression in log2 scale. Positive log2FC indicates upregulated microbiome in chemotherapy-sensitive patients; negative log2FC indicates downregulated microbiome in chemotherapy-sensitive patients.

**Figure 3:**
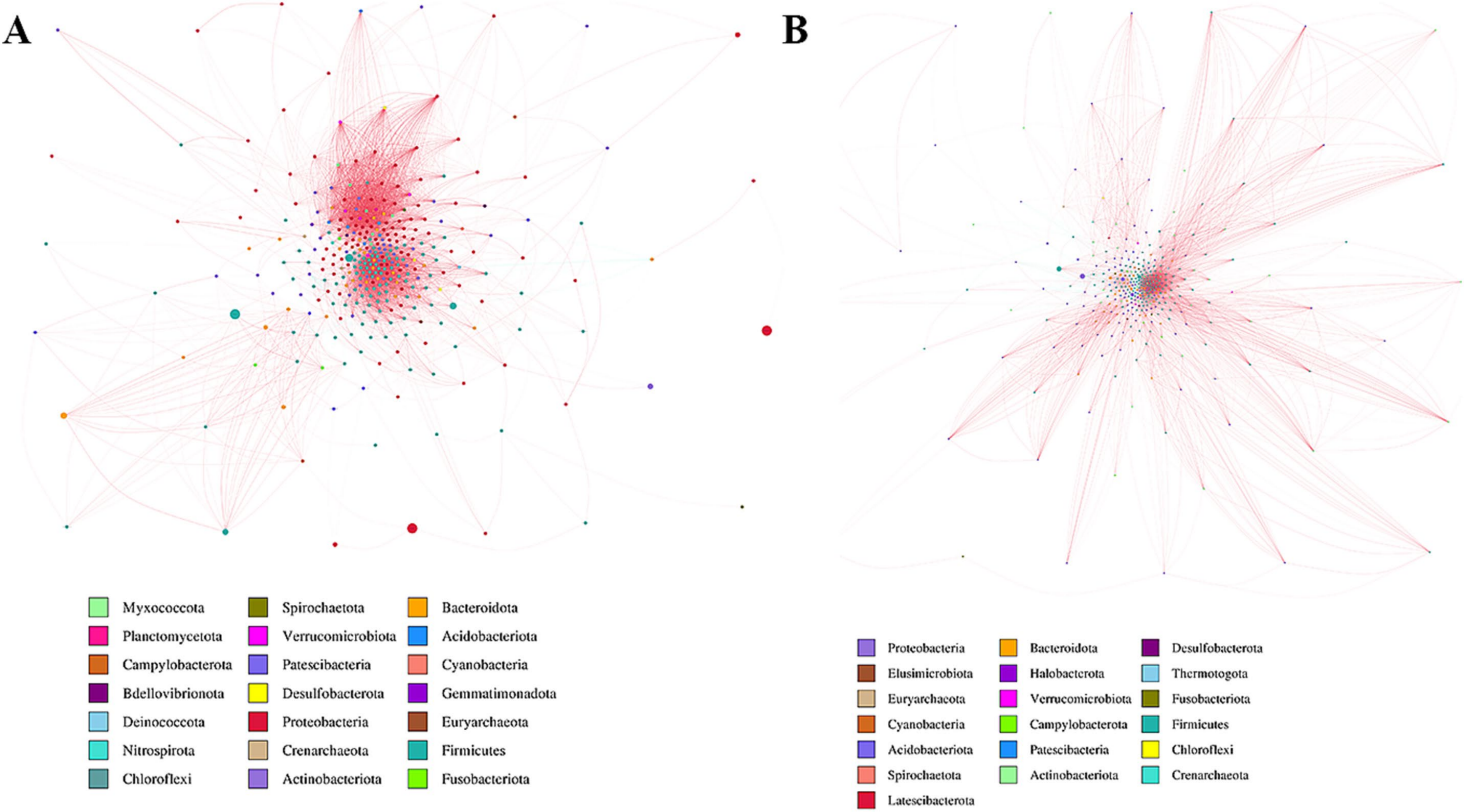
Network analysis of two groups. Potential bacterial interaction was explored by a co-occurrence network analysis, based on the 16S rRNA gene. **(A)** Sensitive group. **(B)** Insensitive group.

The predicted functions of bacteria associated with lung cancer were analyzed utilizing PICRUSt2. Ten key functions were determined to be most significant for lung-associated bacteria, as illustrated in Figure 2F. Among these, ABC transport system-related pathways were significantly clustered (see Figure 3).

### 3.4 Combined analysis of microbiomics and metabolomics

**Figure 4:**
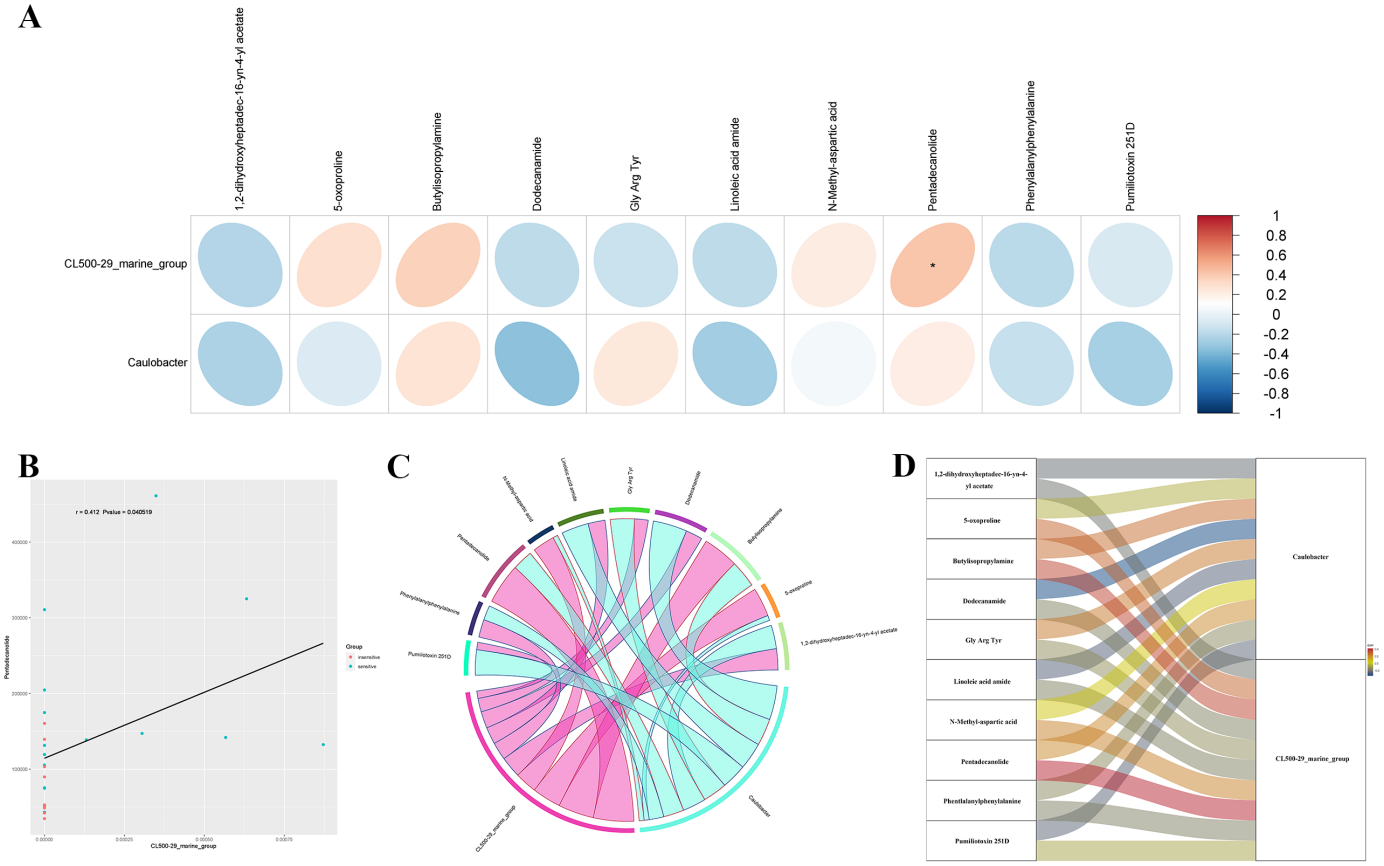
Combined analysis of microbiomics and metabolomics. **(A)** Heatmap analysis of the correlation between significantly different bacterial genera at the genus level and significantly different metabolites obtained by metabolomics analysis. **(B)** Scatter plot of correlation analysis for CL500-29_marine_group and Pentadecanolide. **(C,D)** A chord plot **(C)** and a Sankey diagram **(D)** showing the correlations between key metabolites and key bacterial genera.

To examine the phenotypic modifications that may be elicited by changes in the host’s microbial community structure, we performed an association analysis between the metabolome and the microbiome. Initially, a correlation analysis was performed, and a heatmap was generated using the Pearson correlation coefficient to investigate the significantly different genera identified through 16S rDNA analysis, as well as the significantly different metabolites identified through metabolomics analysis. This study sought to evaluate the degree of association between species diversity and metabolites in environmental samples. The findings revealed a positive correlation between CL500-29_marine_group and Pentadecanolide (*r* = 0.412; *p* = 0.041), both of which were significantly elevated in the alveolar lavage fluid of patients sensitive to chemotherapy (Figures 4A,B).

A chord plot and a Sankey diagram was developed using the correlation coefficients between differential bacterial genera and differential metabolites, providing a visual representation of the associations between key metabolites and key bacterial genera (Figures 4C,D).

### 3.5 Alteration of serum bile acids in lung cancer patients

The findings indicate a correlation between the cholic acid concentration in alveolar lavage fluid and the chemosensitivity of lung cancer. This association is particularly noteworthy given that bile acids are commonly present in the digestive system. Consequently, we conducted a targeted metabolomic analysis of bile acids in pre-chemotherapy blood samples obtained from these patients.

**Figure 5:**
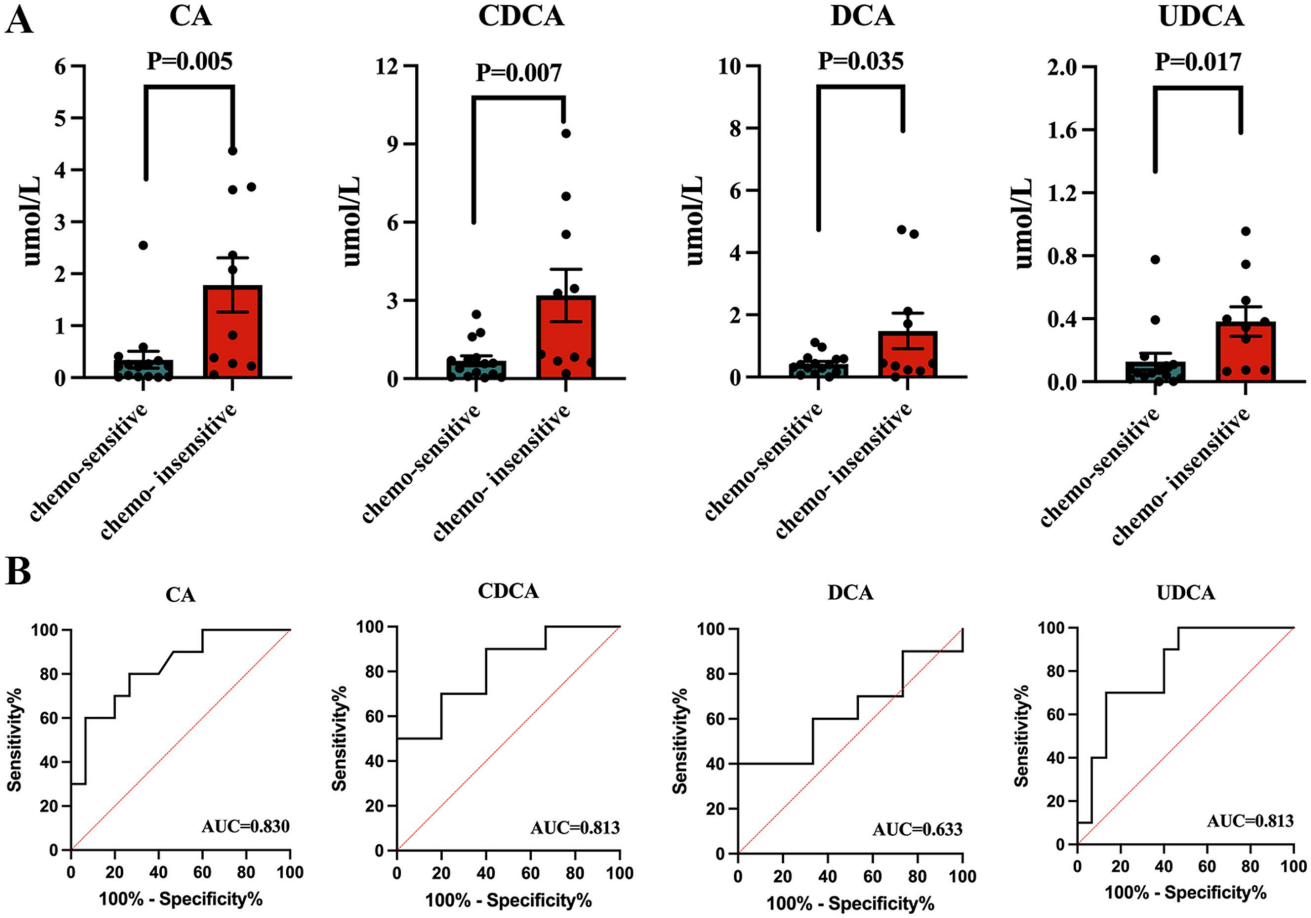
Serum bile acid in lung cancer patients. **(A)** Concentrations of total bile acid, chenodeoxycholic acid, and cholic acid between lung cancer patients and healthy controls. **(B)** ROC curve analysis on the three bile acids to assess their diagnostic value for lung cancer.

The results revealed that the concentrations of four specific bile acids, chenodeoxycholic acid (CDCA), cholic acid (CA), deoxycholic acid (DCA), and ursodeoxycholic acid (UDCA) were notably higher in the chemotherapy sensitive lung cancer patients, with *p*-values of 0.007, 0.005, 0.035, and 0.017, respectively (Figure 5A). No significant difference was found in other bile acids (Supplementary Figure 2). We conducted a ROC curve analysis on the four bile acids to assess their diagnostic value for lung cancer, finding AUC values of 0.830 (CA), 0.813 (CDCA, UDCA), and 0.633 (DCA) (Figure 5B).

## 4 Discussion

The findings of this study provide compelling evidence for the potential roles of the pulmonary microbiota and metabolome in influencing chemotherapy response in lung cancer patients. By integrating microbiomic and metabolomic analyses of BALF, we identified distinct microbial and metabolic signatures associated with chemotherapy sensitivity, offering new avenues for personalized treatment strategies.

Our study identified significant variations in the relative abundances of specific bacterial genera, including Caulobacter, CL500-29_marine_group, and Acinetobacter, between two cohorts. These findings are consistent with emerging literature that underscores the influence of the lung microbiome on immune responses and tumor microenvironments. Specifically, the increased presence of Caulobacter in sensitive patients may indicate its potential role in enhancing drug efficacy, potentially through mechanisms of immune modulation or direct interactions with chemotherapeutic agents. In contrast, the decreased abundance of Acinetobacter in sensitive patients suggests a possible association with resistance mechanisms, necessitating further investigation into its functional impact on treatment outcomes.

The lack of significant differences in alpha and beta diversity between groups suggests that overall microbial community structure may not be the primary determinant of chemotherapy response. Instead, specific taxa and their functional pathways, such as those involved in ABC transport systems, appear to play a more critical role. This observation underscores the importance of moving beyond diversity metrics to focus on functionally relevant microbial features in future studies.

Our metabolomic analysis identified 92 differentially abundant metabolites, with trans-urocanate and phenylalanylphenylalanine exhibiting the most pronounced changes. The elevation of trans-urocanate, a metabolite linked to histidine metabolism, in sensitive patients may reflect its role in anti-inflammatory or immunomodulatory processes, which could enhance chemotherapy efficacy. Conversely, the decrease in phenylalanylphenylalanine might indicate disrupted peptide metabolism in resistant tumors, potentially serving as a biomarker for poor response.

Pathway enrichment analysis highlighted ABC transporters, glutathione metabolism, and bile acid biosynthesis as key pathways associated with chemosensitivity. The involvement of ABC transporters is particularly noteworthy, as these proteins are known to influence drug efflux and resistance. Additionally, the correlation between specific bile acids (e.g., CDCA, CA) and chemosensitivity suggests a novel link between systemic metabolism and lung cancer treatment response. This finding raises intriguing questions about the gut-lung axis and whether bile acids or their microbial derivatives could be harnessed to improve therapeutic outcomes.

The identification of microbial and metabolic biomarkers, such as specific bile acids and bacterial genera, holds promise for developing predictive models to guide chemotherapy decisions. However, the primary limitation of this study is the relatively small cohort size (*n* = 25), which may affect the generalizability of our findings. Larger, multi-center studies are needed to validate the microbial and metabolic signatures identified here. Future research should expand to larger, multi-center cohorts and incorporate functional assays (e.g., *in vitro* or animal models) to validate the mechanistic roles of identified microbes and metabolites.

Moreover, the interplay between the gut and lung microbiomes warrants further exploration, as systemic metabolites like bile acids may originate from gut microbial activity. Investigating whether modulating these microbial or metabolic pathways (e.g., through probiotics, dietary interventions, or targeted therapies) could enhance chemotherapy response represents an exciting frontier in precision oncology.

## 5 Conclusion

This study underscores the potential of integrating microbiomic and metabolomic profiling to unravel the complex determinants of chemotherapy response in lung cancer. By identifying specific microbial and metabolic signatures associated with treatment outcomes, our work lays the foundation for future research aimed at personalizing lung cancer therapy and improving patient survival.

## Data Availability

The datasets presented in this study can be found in online repositories. The names of the repository/repositories and accession number(s) can be found in the article/Supplementary material.
