# Supplementary material for: Pulmonary microbiome and metabolome signatures associate with chemotherapy response in lung cancer patients
Source: Front Microbiol. 2025 Jun 13;16:1604999. doi: 10.3389/fmicb.2025.1604999 (PMC12202651; doi:10.3389/fmicb.2025.1604999)
Supplement: Supplementary file 1 [file Data_Sheet_1.docx]

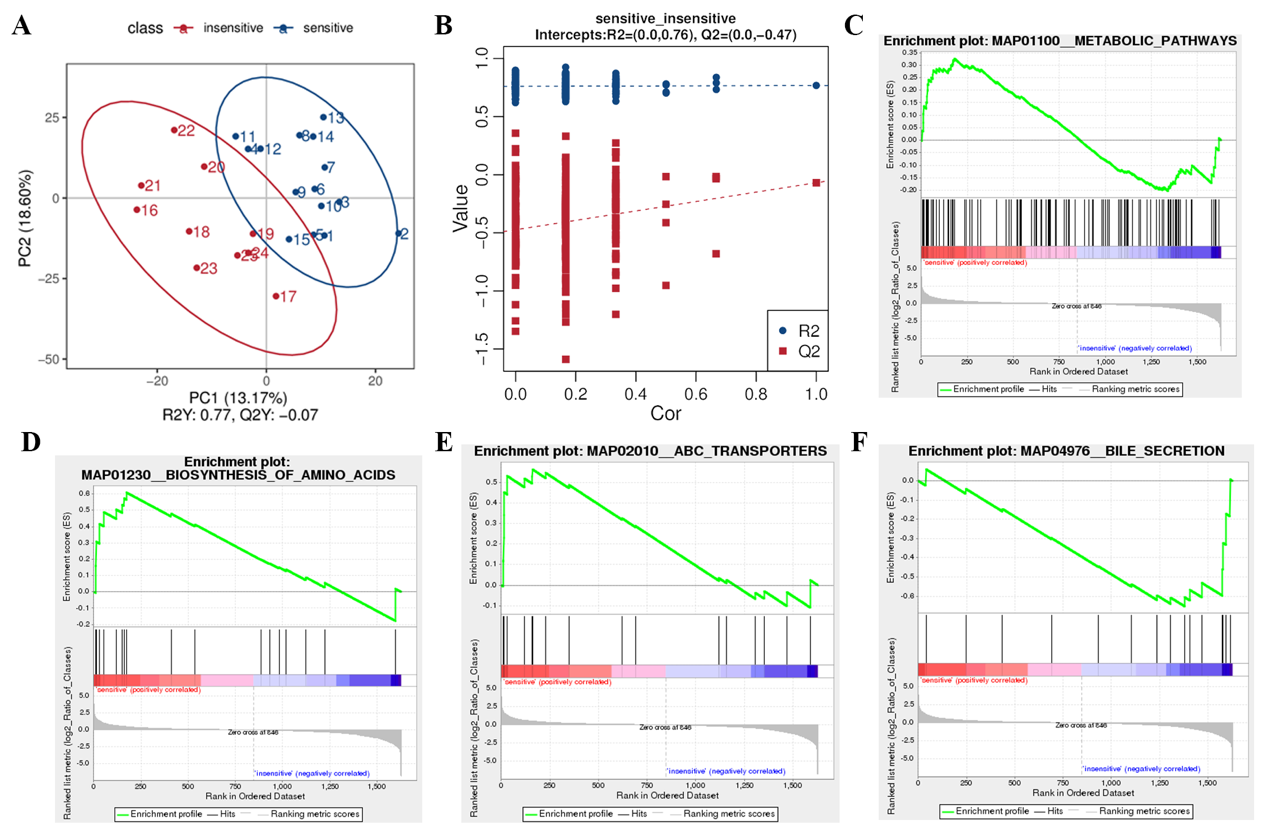


**Supplementary Figure1: A.** Partial Least Squares Discriminant Analysis (PLS-DA) between two groups; **B.** Displacement tests between two groups; **C-F.** GSEA analysis of differential metabolites revealed significant enrichment in Metabolic pathways.

**
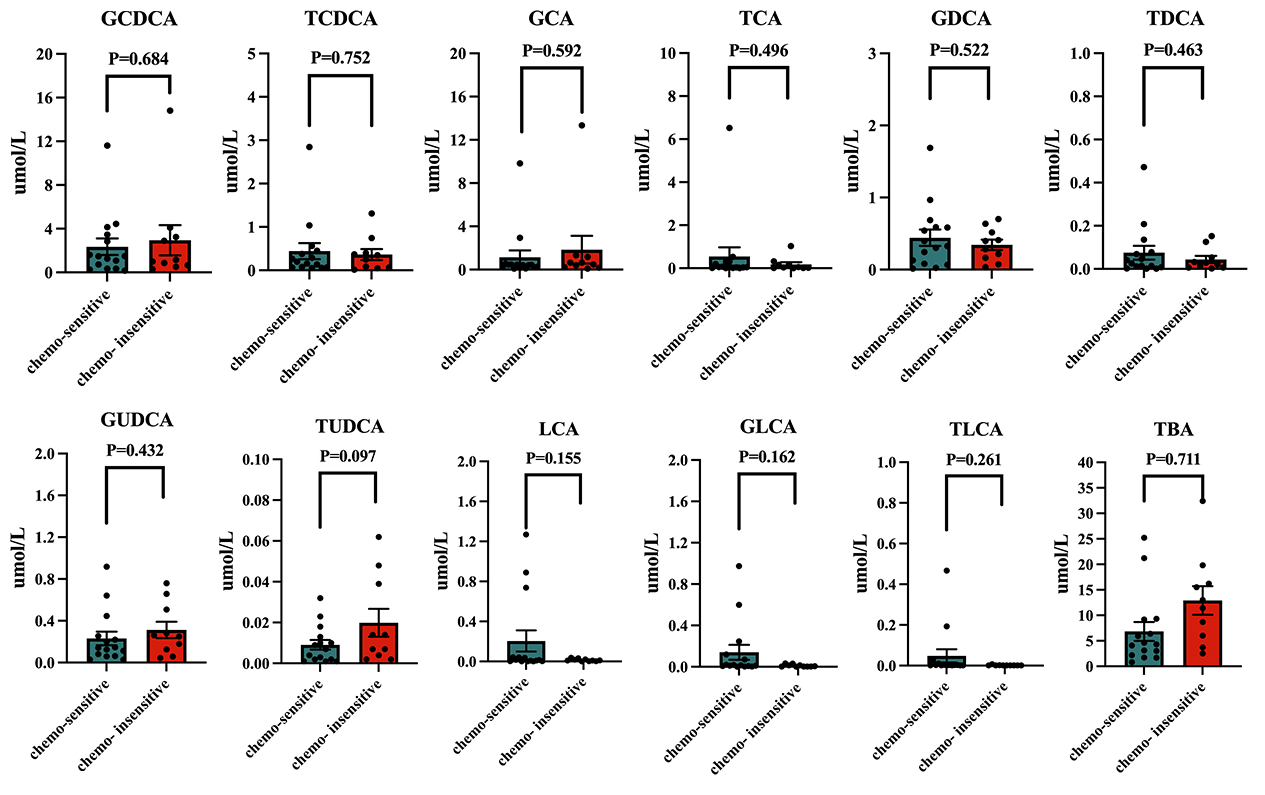
**

**Supplementary Figure2:** No significant difference was found in indicated bile acids between two groups.
